# Contactless, autonomous robotic alignment of optical coherence tomography for in vivo evaluation of diseased retinas

**DOI:** 10.21203/rs.3.rs-2371365/v1

**Published:** 2023-01-09

**Authors:** Ryan McNabb, Pablo Ortiz, Kyung-Min Roh, Ailin Song, Mark Draelos, Stefanie Schuman, Glenn Jaffe, Eleonora Lad, Joseph Izatt, Anthony Kuo

**Affiliations:** Duke University; Duke University; Duke University; Duke University; Duke University; Duke University; Duke University; Duke University; Duke University; Duke Eye Center

## Abstract

During the COVID-19 pandemic, an emphasis was placed on contactless, physical distancing and improved telehealth; contrariwise, standard-of-care ophthalmic imaging of patients required present, trained personnel. Here, we introduce contactless, autonomous robotic alignment of optical coherence tomography (RAOCT) for *in vivo* imaging of retinal disease and compare measured retinal thickness and diagnostic readability to technician operated clinical OCT. In a powered study, we found no statistically significant difference in retinal thickness in both healthy and diseased retinas (*p* > 0.7) or across a variety of demographics (gender, race, and age) between RAOCT and clinical OCT. In a secondary study, a retina specialist labeled a given volume as normal/abnormal. Compared to the clinical diagnostic label, sensitivity/specificity for RAOCT were equal or improved over clinical OCT. Contactless, autonomous RAOCT, that improves upon current clinical OCT, could play a role in both ophthalmic care and non-ophthalmic settings that would benefit from improved eye care.

## Introduction

Examination of the eye is critical for diagnosing vision threatening sequelae of systemic and ocular diseases (1). While the front of the eye is readily accessible visually to an examiner, the back of the eye, where the retina resides, requires specialty diagnostic devices to examine. Eye care specialists use a variety of devices including direct or indirect ophthalmoscopy, fundus photography, and optical coherence tomography (OCT) to examine the retina (2–5). Of these techniques, only OCT provides 3D tomographic, quantitative information (4). A commonality of all these retinal diagnostic techniques is the requirement for physically present, trained personnel to examine and/or image the patient.

With the recent COVID-19 pandemic, an increased emphasis was placed on physical distancing and contactless interactions. These new priorities considerably affect existing clinical processes including the accessibility of all retinal examination techniques. Indeed, during the first year of the pandemic, ophthalmic specialty visits decreased by over 75% compared to the prior pre-pandemic year (6). Again, due in part to the need for physically present, trained personnel to operate retinal diagnostics to directly examine patients, the second lowest utilizer of telemedicine for remote care during the pandemic was the eye care field (with only physical therapy ranking lower) (7).

Robotics have been used in other fields particularly for applications where the physical presence of a person may be unsafe or to allow a person to be “virtually present”, however, only recently have they been introduced for autonomous medical diagnostic imaging purposes (8–11) with only a select few leaving the engineering lab and reaching a clinical population (11). With this in mind, we used a robot equipped with a face and eye tracking vision system (12, 13) as the basis for a physically distanced and contactless retinal examination system that could be used in a clinical environment for a variety of adult patients (i.e., different genders, ethnicities, ages, systemic diseases which can affect patient stability, etc.). Because OCT over the last two decades has increasingly become a mainstay of the modern clinical retinal examination surpassing even fundus photography (14), the OCT modality was incorporated onto the robot for retinal diagnostic capabilities. After development of the system, we imaged a cohort of patients with a variety of retinal diseases who were undergoing their standard of care retinal eye examinations, and we demonstrate that autonomous robotic alignment of OCT was diagnostically comparable to, or better than, current clinically available OCT. The development of this robotically aligned OCT (RAOCT) system has potential utility for retinal examinations both during the current pandemic affected environment and in the post-pandemic future for increasing the accessibility of retinal eye diagnostics without the need for physically present, trained personnel.

## Results

### Autonomous tracking and motion compensation of patients in 3D space

Robotically aligned OCT automatically tracks and compensates for patient motion in near real-time utilizing tracking cameras combined with open and closed loop control of the robot tool end effector and electronically controlled opto-mechanics (see Methods) (12). Face-tracking cameras and software identified the location of the patient’s face and tracked the target eye of the patient. Using an open loop control system, these estimated coordinates were used to direct the robot to automatically align to the patient’s eye and compensate for bulk patient motion during imaging. Once grossly aligned, pupil-tracking cameras tracked the target ocular pupil of the patient. Using a closed loop control system, these residual position estimates were used to position the robot, sample arm 2D scanning mirror, and voice-coil reference arm. This negated the requirement for patient stabilization, such as chin and forehead rests, during image acquisition.

**Figure 1:**
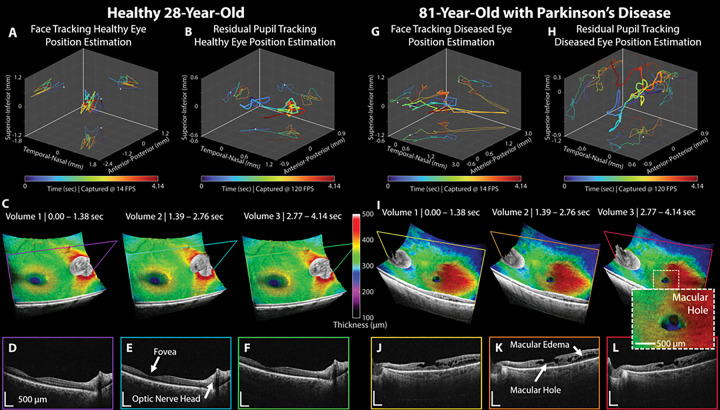
Robotically aligned retinal OCT active motion compensation and volume acquisition in healthy patient and diseased retina from Parkinson’s patient with tremor. Healthy patient and retina: **(A)** Face-tracking estimation of right eye motion during three-volume (4.14 sec) OCT acquisition of healthy retina. The reference origin (0, 0, 0) mm is shown in white. Motion in a given 2D plane is projected on to the corresponding edge plane. **(B)** Residual motion estimation from pupil-tracking of right eye. **(C)** Three OCT volumes in a sequentially acquired time-series showing little variability between volume acquisitions. Retinal thickness (μm), from inner limiting membrane (ILM) to retinal pigment epithelium (RPE), was mapped to the top surface of the OCT volume. The RPE does not continue through the optic nerve head (ONH), and thus retinal thickness was excluded in the area surrounding the ONH. **(D-F)**Individual B-scans from volumes in (C). Parkinson’s patient: **(G)** Face-tracking estimation of left eye motion during three-volume (4.14 sec) OCT acquisition of diseased retina. In addition to diseased retina, this patient had tremors due to Parkinson’s disease greatly increasing the amount of motion relative to the healthy patient. **(H)** Residual motion estimation from pupil-tracking of left eye. High frequency residual error, due to patient tremors, remains following face-tracking. **(I)** Three OCT volumes in a sequentially acquired time-series with thickness maps. Even with motion due to tremors, the resulting volumes show little variability during and between acquisitions. Final volume inset illustrates benefits of active motion compensation with a detailed view of macular hole processes. **(J-L)**Individual B-scans from volumes in (I) showing macular hole and macular edema.

Figure 1 demonstrates the benefits of active motion compensation through estimated motion profiles and corrected OCT volumes in two different patients: one with a healthy retina and the other with both a diseased retina and tremors due to Parkinson’s disease. Tracking profiles illustrate motion estimation in three dimensions: Temporal-Nasal (*T-N*, fast OCT dimension), Superior-Inferior (*S-I*, slowOCT dimension), and Anterior-Posterior (*A-P*, depth OCT dimension). For both patients we show concurrent face-tracking and residual pupil-tracking position estimation and three consecutive OCT volumes acquired over a total of 4.14 seconds. Face-tracking of a healthy retina patient (Fig. 1A, undialated, 28 year old female; see Methods, Study Design) indicates a preliminary motion estimation of the right eye over a range of (1.5, 0.7, 2.1) mm [*T-N*, *S-I*, *A-P*]. Pupil-tracking (Fig. 1B) estimated a position residual range of (1.5, 0.4, 0.9) mm. In contrast, for the patient with tremors due to Parkinson’s disease (Figs. 1G&H, 81 year old female), face-tracking estimated a range of (2.5, 1.0, 3.4) mm and pupil-tracking estimated a position residual of (1.5, 0.9, 1.8) mm for their left eye. Our face-tracking algorithm estimates the ocular globe location and does not track the ocular pupil location. Pupil-tracking residual values account for both eye motion that occurs during image acquisition and residual robotic position error due to open loop control.

For the healthy patient, Figs.1C–F show three RAOCT volumes and corresponding B-scans acquired during the motion profile time sequences shown in Figs. 1A&B. All three volumes provide a stable, clinically relevant field-of-view (FOV) with both the foveal pit and optic nerve head (ONH) visible. Retinal thickness (μm), here defined as the thickness between the inner limiting membrane (ILM; inner most layer of the retina before vitreous) and retinal pigment epithelium (RPE; hyper-reflective pigmented epithelial layer at outer boundary of neurosensory retina and before Bruch’s membrane and choroidal vasculature), was mapped to the top surface of the OCT volume. We automatically excluded the area surrounding the ONH as the RPE and the corresponding retinal tissue do not continue through the ONH and represents the ‘blind-spot’ in an individual’s vision. The foveal pit thickness in this patient measured 280 μm (mapped to blue) while thickening toward ONH to 450 μm (mapped to red) as the nerve fiber layer increased in thickness.

For the patient with diseased retina and tremors, Figs. 1I–L show three RAOCT volumes and corresponding B-scans acquired during the motion profile time sequences shown in Figs. 1G&H. All three volumes show a stable, clinically relevant FOV however unlike the healthy subject, the foveal pit is not readily visible due to macular edema (mapped to red) and the presence of a macular hole (mapped to blue; detail in inset). Additionally, volume three (B-scan in Fig. 1L) shows a small nasal shift in FOV likely due to uncorrected changes in patient gaze. However, while there was millimeter scale motion of the patient during OCT volume acquisition, two-tiered tracking and compensation allow for visualization of the 50 μm walls of the macular hole processes (Fig. I, inset).

### Retinal imaging with robotically aligned optical coherence tomography

**Figure 2:**
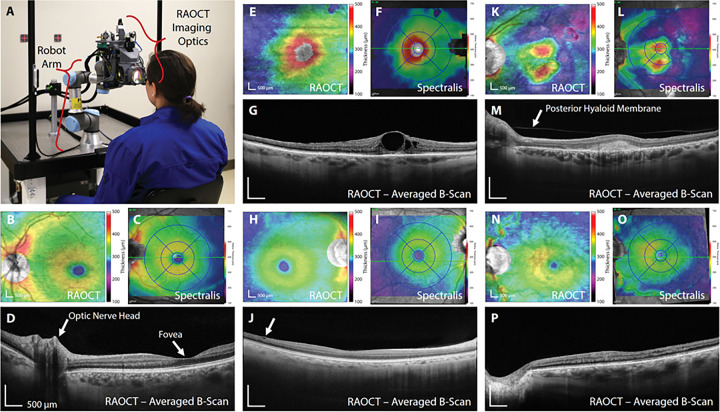
Clinical robotically aligned OCT retinal imaging with thickness map comparision to commercial SDOCT imaging (Heidelberg Spectralis) utilizing the same colormap. **(A)** Photograph of RAOCT system imaging patient with fixation targets in background. B-scan acquired during photograph shown in (D). **(B-D)** Healthy left retina of 26 yo patient; RAOCT and Spectralis thickness maps with RAOCT averaged B-scan showing foveal pit and edge of optic nerve head. RAOCT thickness maps are overlaid on summed volume projections from the original OCT data. Spectralis thickness maps are overlaid on separately acquired, larger FOV scanning laser ophthalmoscopy images. **(E-G)** Right retina of 81 yo woman with panuvetis resulting in cystoid macular edema. **(H-J)** 51 yo woman with mild epiretinal membrane with minimal traction resulting in a hyporeflective space (arrow) and systoid change in retinal surface. **(K-M)** Left retina of 43 yo woman with history of bilateral multifocal choroiditis and panuveitis syndrome with optic nerve drusen and choroidal neovascular membrane **(N-P)** Left retina of 66 yo man with severe glaucoma and central retinal vein occlusion with resolving cystoid macular edema.

To quantitatively evaluate RAOCT, we imaged patients with healthy and diseased retinas recruited from clinics at the Duke Eye Center and compared the RAOCT measurements with clincially available, technician acquired OCT. We imaged each subject in triplicate with RAOCT and a clinical spectral-domain OCT system, OCT Spectralis from Heidelberg Engineering (see Methods, Study Design). For each acquired RAOCT volume we generated a retinal thickness map (from ILM to RPE) which matches the tissue boundaries utilized by Spectralis to generate their thickness maps (15). Representative thickness maps from both devices in healthy and diseased retinas can be seen in Fig. 2. It should be noted that, RAOCT thickness maps were overlaid on summed volume projections from the RAOCT data utilized to generate the thickness maps while the Spectralis thickness maps were overlaid on separately acquired, slightly larger FOV scanning laser ophthalmoscopy (SLO) images. Autonomous robotic alignment and compensation allows for the patient to sit without chin or forehead rests as seen in Fig. 2A. Taking advantage of active compensation, we acquired, registered, and averaged up to 100 repeated B-scans at a single location enabling high resolution retinal images. A healthy retina, as seen in Fig. 2D, shows the fovea (the region of retina responisble for our highest acuity vision) and vasculature surrounding the optic nerve head. This particular B-scan was acquired during the photograph shown in Fig. 2A. When imaging diseased retina, fine details such as walls of cystoid macular edema (Fig. 2G) and the boundary of the posterior hyaloid membrane (Fig. 2M) can be seen.

**Figure 3:**
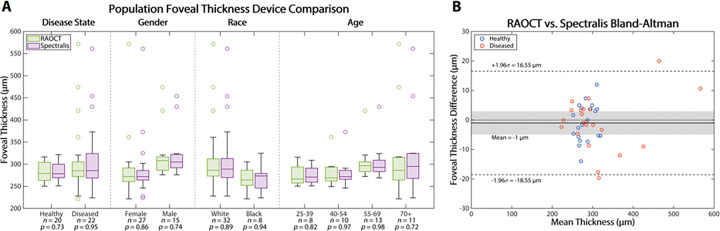
Clinical robotically aligned OCT retinal imaging with thickness map comparision to commercial SDOCT imaging (Heidelberg Spectralis). **(A)** Box plot comparing central foveal thickness as measured by RAOCT and Spectralis across a variety of demographics. There was no stastisitically significant difference between devices for any investigated demographic. **(B)** Bland-Altman difference plot comparing both devices in healthy and diseased retinas.

To quantitatively compare the two devices, we calculated the mean retinal thickness in a 1 mm diameter area centered at the fovea for each acquired volume. This metric is important because changes in foveal thickness, and macular thickness overall, are relevent biomarkers for diabetic retinopathy (16, 17) and macular degeneration progression(18–20); specifically, retinal pathologies can cause retinal thickness increases (e.g., from edema) or decreases (e.g., from atrophy or degeneration). We imaged each eye in triplicate to investigate intra-session variation. In Table 1, we report mean foveal thickness across the three volumes and intra-session variation. Several patients with diseased retinas had one of their eyes excluded from the study due to comorbidities (i.e. cataract, tremors, enuculation) that prevented the technician from acquiring volumes with clinical OCT. For RAOCT, the mean foveal thicknessacross all eyes was 297.8 μm with a mean intra-session variation of ±1.5 μm and with a total population variation of ±61.7 μm. We then split the population into healthy and diseased retinas. For healthy retinas, the mean thickness was 282.9 μm, intra-session variation was ±0.8 μm, and with a total population variation of ±21.7μm. For diseased retinas, the mean thickness was 310.3 μm, intra-session variation was ±2.0 μm, and with a total population variation of ±79.1 μm. For clincial OCT, the mean foveal thicknessacross all eyes was 297.9 μm with a mean intra-session variation of ±7.0 μm and with a total population variation of ±59.8 μm. For healthy retinas, the mean thickness was 283.7 μm, intra-session variation was ±2.17 μm, and with a total population variation of ±19.8 μm. For diseased retinas, the mean thickness was 310.8 μm, intra-session variation was ±11.4 μm, and with a total population variation of ±78.3 μm. When comparing the pair-wise difference in measurements between devices (see Fig. 3A) there was no statistically significant difference in central foveal thickness measurements between RAOCT and current, technician acquired clinical OCT for neither healthy retinas (*p* = 0.73, ICC = 0.956 [0.894 – 0.982]) nor diseased retinas (*p* = 0.95, ICC = 0.994 [0.986 – 0.998]). These limited differences can be better illustrated in the Bland-Altman plot in Figure 3B where there was a mean thickness difference of −1 μm between devices across the entire imaged population.

To establish that RAOCT performs comparably to clinical OCT across a broad range of demographics, we combined all thickness measurements and re-evaluated measured foveal thickness based on gender (female and male), race (White and Black), and age (25 – 39 years old, 40 – 54, 55 – 69, and 70 and older). Note that because we were only able to image a single patient of Asian descent, this individual was excluded from device comparisons for the race categorizations only. When comparing devices based on gender: female, *p* = 0.86 ICC = 0.994 [0.987 – 0.997] and male, *p* = 0.74 ICC = 0.986 [0.960 – 0.995]. When comparing devices based on race: White, *p* = 0.89 ICC = 0.994 [0.988 – 0.997] and Black, *p* = 0.94 ICC = 0.960 [0.830 – 0.992]. When comparing devices based on age: 25–39 years old, *p* = 0.82 ICC = 0.943 [0.767 – 0.988], 40 – 54 years old, *p* = 0.97 ICC = 0.985 [0.944 – 0.996], 55 – 69 years old, *p* = 0.98 ICC = 0.985 [0.955 – 0.996] and 70 and older, *p* = 0.72 ICC = 0.996 [0.984 – 0.999]. Importantly, when comparing the pair-wise difference in measurements between devices, we found no statistically significant difference in any of the sub-catagorizations with all *p*-values greater than 0.7 and ICC values greater than 0.9.

### Clinical diagnostic comparison

In addition to providing quantitative information, OCT images are utilized in the clinic for diagnostic purposes. To test the diagnostic utility of the images, we performed a secondary study using the acquired patient data wherein a retina clinician experienced with OCT interpretation labeled a given volume as either normal or abnormal and compared the results to the clinical diagnostic label (see Methods, Study Design). We found the sensitivity and specificity for detecting abnormal retinas to be 93% and 90%, respectively, for RAOCT and 87% and 60% for Spectralis. We found the positive and negative predictive values of RAOCT to be 93% and 90% while they were 76% and 75% for Spectralis.

## Discussion

In this work, we demonstrate autonomous robotic alignment of an OCT system in a retinal clinic population and compare its use to a clinical OCT system. For both quantitative and qualitative metrics, we found that RAOCT met or exceeded the performance of the clinical OCT system. Quantitatively, there was no statistically significant difference between central foveal thickness measurements for any population group with a mean difference of −1 μm between the devices, however, RAOCT consistently provided better repeatability through lower intra-subject variation between measured volumes. Qualitatively, when a trained grader evaluated these retinal volumes as abnormal or normal, the sensitivity, specificity, PPV, and NPV were at least as high as the clinical OCT system.

Autonomous robotic alignment removes the requirement for a trained ophthalmic photographer, optometrist, or ophthalmologist to acquire clinically useful OCT images. While there is value in having robotic imaging at a tertiary care facility, an important potential benefit of such as system comes when placed in more remote locations with less specialized personnel resources such as rural hospitals and primary care locations. Prior works using non-specialized personnel to perform retinal screening with other imaging techniques (e.g. fundus photography) have suffered from a high number of poor quality or ungradable images due in part to poor ocular alignment and focusing by the non-specialized operator (21, 22). With our RAOCT system, local alignment was autonomous and thus the specialized operator could be remotely located. This means that locally (i.e. location of robot and patient), non-specialized personnel act only as chaperones to place the patient in front of the RAOCT system. RAOCT autonomously aligns to the patient’s eye and maintains alignment; the centralized remote operator would need only to confirm image quality, make adjustments to target a region of interest (13), and trigger the acquisition once a high quality image is present. The remote operation also presents potential for telehealth opportunities. Additionally, because OCT was not part of our robotic vision system, autonomous robotic alignment could be utilized for other imaging technologies including for non-ophthalmic applications (11).

Active compensation for patient motion can benefit current ophthalmic clinics as well. As an example, we did not report central foveal thickness for the left eye of Patient 18 (Table 1) because the technician was unable to acquire Spectralis OCT volumes on this patient due to tremors from Parkinson’s disease. The motion compensated RAOCT volumes (and motion profiles) from the left eye of this patient are shown in Figure 1G–L, revealing details of the present macular hole despite large amounts of eye motion. While this degree of compensation is helpful, introduction of active gaze compensation could further increase the usefulness of RAOCT in harder to image populations such as those with nystagmus (a type of involuntary, rapid, and undesired motion of the eye) or in pediatric ophthalmology.

In addition to comparing patients with healthy and diseased retinas, we performed a secondary analysis of our measurements that demonstrated invariance to a range of demographics. There have been numerous instances of algorithms exhibiting a preference for individuals with lighter skin tones, and it was important as a potentially screening technology that RAOCT instead be agnostic. During our initial system development and prior to this study, we noted that the LEDs used for pupil-tracking were causing a bright spot to appear in the center of the face on people with higher melanin concentrations when viewed through the face-tracking cameras (23). We introduced a short-pass optical filter to the face-tracking cameras to resolve this issue (See Methods, Clinical robotically aligned optical coherence tomography system design). During the above study, skin tone never limited our ability to image an individual but this does highlight the need for studies regarding the use of image-guided systems across a broad range of demographics, including but not limited to race, prior to general clinical use.

Robot-patient interaction and overall human safety were high priorities during our system and experiment design. However, we did not fully anticipate how the patient would interact with the robot. Anecdotally, most patient responses were quite positive commenting that they were “living in the future,” or quickly personalizing the robot and wanting to know its name (‘Bernard’). Conversely, a small minority of patients were reticent and expressed concerns about robots in general or the idea of face-tracking. It should be noted that the face-tracking algorithm we utilized detects objects within a scene that it identifies as a face and not that a face belongs to a given individual (24). As robots become more commonplace in the clinic, there is a need to study human-machine interactions in this specialized environment.

We statistically powered our study to detect the measured central foveal thickness difference between two OCT devices in a healthy population. While the mean thickness difference between devices was small (−1 μm), our system consistently reported lower intra-subject variation. Ideally, this lower variation would be indicative of the value of autonomous alignment and indeed might be. However, there are two other possible explanations: scan density and manual foveal selection. We designed our volumetric scan pattern to be approximately equal in acquisition time to the one used by Spectralis. However, our OCT engine acquires data at 2.5x the rate of the Spectralis system (100k A-scans/sec vs 40k A-scans/sec), and as a result, we captured higher density volumes with an emphasis on increasing the sampling along the superior-inferior direction and thus increasing the number of B-scans per volume. Given that we utilized more B-scans per volume and that neither system critically samples the retina in this direction, we were more likely to capture the true foveal center for any given volume acquisition. Selecting the foveal center in a given volume was performed manually in RAOCT (with semi-automatic retinal thickness segmentation) and automatically on Spectralis volumes (with automatic segmentation and as part of the Spectralis imaging software). Given the precision of automated algorithms on repeated measurements we did not initially consider manual selection to be an advantage (15). However, it is possible that selection bias could be introduced if all retinal thickness maps acquired in triplicate were viewed simultaneously or in quick succession while manually locating the fovea, even though this was not part of the selection protocol.

In this study, we demonstrate that RAOCT was capable of both quantitative and qualitative imaging of retinal clinic patients and that those results were comparable to a current clinical OCT system. This represents one of the first uses of autonomous robotic alignment for medical diagnostic imaging in any clinical setting (11). This study was performed in an academic medical center and is likely better resourced than the majority of eye care clinics, particularly community-based ones. To make this type of system more generally available beyond a major academic medical center, commercial industrial and mechanical engineering design would be beneficial to enhance system robustness to meet the needs of a busy ophthalmic clinic. Dedicated, larger scale multi-center studies evaluating system performance with adequate demographic and disease representation would be necessary to obtain regulatory approval from governmental oversight agencies). Accomplishing these steps would potentially allow RAOCT to play a larger role in ophthalmic care and potentially even into non-ophthalmic settings that would benefit from improved eye care diagnostics such as emergency or primary care facilities (25, 26).

## Methods

### Study Design

Informed consent was obtained from all patients under a Duke University Health System Institutional Review Board approved protocol prior to any imaging. Utilizing RAOCT and OCT Spectralis (Heidelberg Engineering), we imaged 10 patients with healthy retinas and 15 patients with diseased retinas for a total population of 25 (18 female, 9 male; age range: 25–91 years, median age: 46 years; 18 White, 5 Black, 1 Asian-descent). When possible, we imaged both eyes of each patient. The RAOCT scanning protocol consisted of 30° × 30° FOV rectangular volumes (900 A-scans × 125 B-scans; 100kHz A-scan rate) and 30° width repeated B-scans (2000 A-scans × 100 B-scans). All RAOCT imaging adheared to the limits outlined in ANSI Z80.36 – 2016. The Heidelberg Spectralis scanning protocol consisted of 30° × 30° FOV rectangular volumes (768 A-scans × 61 B-scans; 40kHz). We designed the RAOCT scan protocol to closely match the Spectralis acquisition time (~1.2 seconds) instead of scan density to maintain similar susceptability to patient motion. To test for intra-patient foveal thickness measurement variation, we acquired OCT volumes for both modalities in triplicate. In the case of RAOCT, the robot engaged the patient, an OCT volume was captured, and the robot was disengaged for each of the three captured volumes.

When imaging with RAOCT, subjects were seated, though unlike conventional OCT imaging, no chin or forehead rests were provided or required. During RAOCT imaging, they were asked to fixate on a target reticle behind the robot. Due to COVID-19 safety protocols, the imaging room (located at Duke Eye Center) was configured such that the operator was greater than 2 meters from the subject, behind a Plexiglas barrier, and wearing an N95 mask. Additionally, room air-quality was improved by two HEPA filters, one near the subject and one near the imager. Due to limitations in our face-tracking algorithm (24), the patients were asked to remove their mask during imaging. For Spectralis, patients were seated in a forehead and chin rest in the ophthalmic photogrpahy suite at the Duke Eye Center. Patients were not dilated as part of our imaging protocol though some individuals received dilation as part of their standard of care during their clinical visit. The dilation state for a given patient was the same when imaged by both RAOCT and Spectralis.

We performed an additional study comparing the ability of a retinal clinician to diagnose retinas as healthy or diseased between both devices. A total of 25 volumes (one from each subject; 15 diseased, 10 healthy) from each device were graded. Grading was done in two sessions, one week apart with one device graded per session. The grader was blinded to the device in a given session. The order of graded volumes was randomized during each grading session. We compared graded the diagnostics to those from the patient’s clinical record to determine sensativity, specificity, positive predictive values, and negative predictive values.

### Clinical robotically aligned optical coherence tomography system design

**Figure 5:**
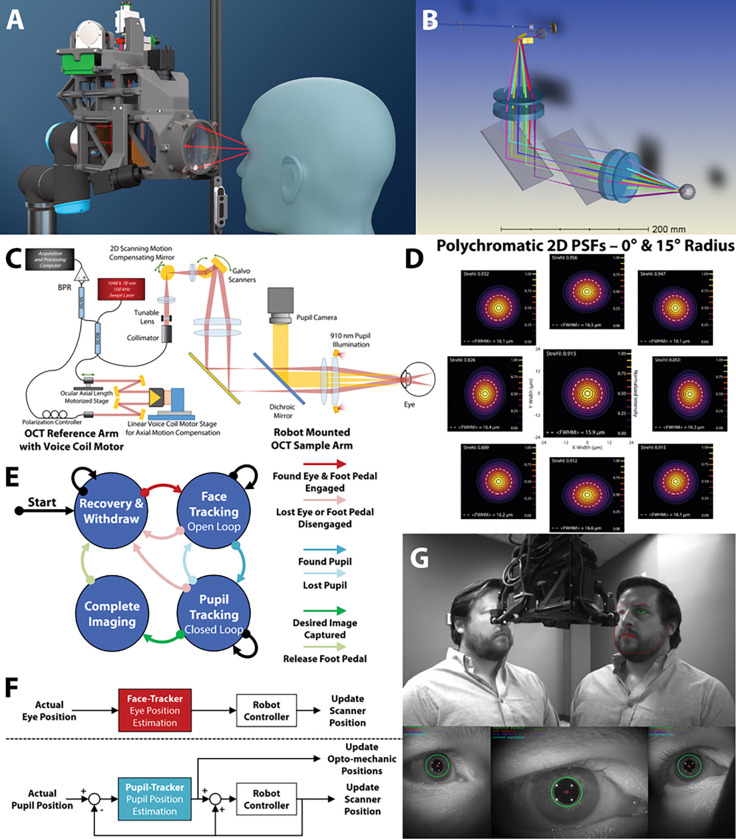
Clinical robotically aligned OCT system design **(A)** Solidworks rendering showing robot mounted OCT imaging system aligned to eye. **(B)** OpticStudio rendering and ray trace diagram with multiple galvanometer scan positions. **(C)** OCT system optical schematic with source, fiber interferometer, sample arm, reference arm, and detection. **(D)**Huygens 2D point spread functions (PSFs) of clinical RAOCT aligned to the optical axis of model eye with scanning mirrors at central position (0°, 0°) and at multiple angles at a 15° radius. System was diffraction limited at all points with a Full Width Half Max (FWHM) spot size of 15.9 – 16.5 μm and a Strehl ratio of >0.825. **(E)** State diagram for two-tiered robotic and OCT system alignment during imaging. **(F)** Open loop eye position and closed loop pupil position systems based on face-tracking and pupil-tracking estimations for updating scanner and opto-mechanic positions. **(G)** Simultaneous views from face and pupil-tracking cameras with tracking enabled and both face and pupil located.

We previously developed a robotically tracking and aligning OCT system that automatically acquired retinal OCT volumes in young, healthy volunteers with ~16° field-of-view (FOV) that was limited by robot payload considerations and working distance safety restrictions (12). Here we report the development of a contactless and autonomously aligned robotic OCT system designed specifically for imaging the diverse population found within retinal ophthalmology clinics (Fig. 5). This system provides a clinically relevant retinal FOV (~32°) providing volumetric images with a motion stabilized view of the fovea and optic nerve head [ONH]) while the autonomous alignment allowed the system operator to maintain a safe distance from the patient due to COVID-19 safety protocols.

Our robotically aligning (Universal Robots UR3e) swept source retinal OCT system (Axsun; λ0=1043nm±72nm; 100 kHz), was designed with custom optics and custom opto-mechanics to simultaneously meet our patient imaging requirements and remain within the mass limitations of the robot. Given a robot safety working distance of 86 mm, a desired retinal FOV of at least 30°, and additional imaging aperture for high speed pupil position compensation, we designed our imaging lenses to be 70 mm in diameter. To minimize mass and cost, we designed an air-spaced achromatic lens and configured a pair of them in a 1:1 4F telescope configuration. Compared to our previous design, we further reduced the mass of our system by utilizing a 15 mm diameter 2D scanning mirror (Optotune MR-E-1) located prior to the galvo scanning mirror pair and conjugate to the retinal imaging plane for active lateral motion compensation(12, 13, 27). Diopter focus control was provided by a 3 mm diameter tunable lens (Optotune EL-3-10-NIR) (28) placed between the laser collimating lens and the 2D scanning mirror telescope. Using the Polans eye model (29, 30), the OCT PSF was designed to be 15.0–17.0 μm FWHM across the full retinal FOV (Fig. 5C–E). Custom opto-mechanics were designed to minimize mass through the use of 3D printing (Formlabs Form3) and carbon fiber cage structure (Fig. 5A). The mass for the OCT imaging module was 2.41 kg and 2.80 kg including cables, both below the 3 kg mass limit of the robot. Our OCT engine utilized a Mach-Zehnder fiber interferometer with transmissive reference arm. We utilized two stages in the reference arm to adjust for the dynamic optical path length of the OCT imaging arm. We accounted for patient ocular axial length by placing one collimator on a linear, motorized translation stage (National Aperture Instruments) which remained fixed during imaging. For active axial motion compensation, we utilized a retroreflector mounted to a linear voice coil motor (VCM; H2W Technologies, VCS05-060-LB-01-MCH) whose position was updated based on pupil location as estimated by the pupil-tracking cameras(Fig. 5B) (12, 31, 32).

Autonomous tracking and compensation of the patient’s eye (Fig. 5F&G) was facilitated through the use of two calibrated face-tracking cameras (Intel RealSense D415; camera module integrated 850 nm and visible room light illumination) and three calibrated pupil-tracking cameras (FLIR Blackfly S BFS-U3-042M; 910 nm LED illumination integrated into OCT imaging opto-mechanics) (12). We specifically chose the wavelength of these pupil-tracking LEDs to mitigate the effects of melanin in the ocular iris so that pupil detection was similar in both brown and blue irises (23, 33). However, when combined with visible room light, these LEDs had the secondary effect of creating high and low reflectivity areas on faces with higher melanin concentrations as observed by the face-tracking cameras. To compensate, we modified the face-tracking cameras include a band-pass optical filter (Semrock, FF01-835/70) to eliminate exposure from the longer NIR wavelengths from both the pupil-tracking camera LEDs and the OCT scanning beam. Without the filters, the ability of our face-tracking algorithm to differentiate a person’s face within a scene was limited (24).

The face-tracking cameras provided initial robot alignment to the target eye of the patient. Utilizing OpenCV and OpenFace face-tracking(24), the position of the target eye was triangulated within 3D space at a rate of 14 frames per second (FPS). An open loop control system was used to translate the tool end effector of the robot and the OCT imaging system to the target eye such that the ocular pupil was in view of the pupil-tracking cameras (Fig. 5H). When in range, the target pupil was viewed simultaneously by all three pupil cameras (Fig. 5F). Binary morphology was utilized to segment and report the centroid of the ocular pupil in each image at 120 FPS (27). The triangulated pupil centroid was used as an input to a closed loop control system (Fig. 5H) to update robot alignment, the 2D scanning mirror position in the OCT sample arm, and VCM position in the reference arm (12).

### Image processing and quantification

Retinal thickness for both RAOCT and Spectralis OCT volumes corresponded to the difference between the retinal inner limiting membrane (ILM) to the retinal pigment epithelium (RPE) (15, 34). For RAOCT, we utilized our previously described semi-automatic, graph cut based segmentation algorithm to determine the pixel difference between layers in a given B-scan (35). That pixel difference was then scaled by the imaging depth in air divided by the group refractive index of retina (*n*_*g_retina*_ = 1.37) to convert pixels to microns (36, 37). We calculated the retinal thickness for all B-scans in a volume. We utilized the retinal thickness values (from ILM to RPE) provided by Heidelberg software for Spectralis OCT volumes. We used the mean retinal thickness in the central 1 mm foveal area for all quantitative analyses.

Data acquired with the RAOCT repeated B-scan protocol were registered and averaged utilizing a pyramidal, non-rigid SURF based registration algorithm (38). From a repeated B-scan stack, we grouped individual frames into smaller, temporally adjacent stacks; for this protocol, every four consecutive frames were grouped such that 100 frames became 25 smaller groups. These small groups were each registered using SURF and averaged to create higher contrast B-scans. These averaged B-scans were manually inspected to remove failed registrations (i.e. subject blinked or large eye motion). We then registered and averaged the remaining averaged B-scans. In this way, up to 100 repeated B-scans could be registered and averaged, as shown in Fig. 2. This technique was not utilized on the OCT volumes used for quantitative analysis or diagnostic quality and only for qualitative display of registered and averaged B-scans (see Fig. 2).

### Statistical Analysis

We imaged 42 eyes from 25 volunteers with *N* = 20 healthy eyes and *N* = 22 diseased eyes with both RAOCT and Heidelberg Spectralis. For each eye, we acquired OCT volumes in triplicate with each device for intra-session repeatability. We powered our study to detect a pair-wise difference in central foveal thickness of 15 μm between RAOCT and Heidelberg Spectralis in healthy retinas (normal-based 95% CI: mean ± 2SD; power = 90%; α = 0.05). This resulted in a target of 18 eyes with an additional two eyes imaged as 10% overhead (20 eyes). Because we imaged a wide array of diseased retinas, we matched the total number of healthy retinas with an additional 10% imaged (22 eyes). We performed 2-sided Wilcoxon signed rank tests comparing mean retinal thickness on both populations. Additionally, we performed 2-sided Wilcoxon signed rank tests when combining both healthy and diseased populations and re-dividing them into populations based on gender, race, and age. We performed pairwise intraclass correlation with upper and lower bounds for all population groupings.

## Figures and Tables

**Figure 4:**
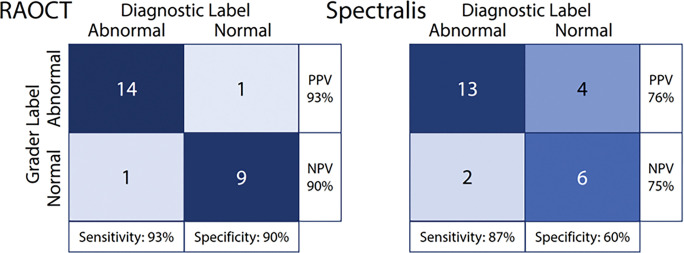
Comparison of grader labeling to clinical diagnosis for both RAOCT and Heidelberg Spectralis with sensitivity, specificity, positive predictive value (PPV), and negative preditive value (NPV) shown.

**Table 1:** Individual central foveal thickness for RAOCT and Heidelberg Spectralis devices in patients with and without retinal disease. Thickness was calculated in the central 1 mm diameter region of the fovea for each eye imaged with both devices in triplicate. Standard deviation represents intra-subject imaging session variation.

**Patients with Healthy Retinas**				**RAOCT Foveal Thickness (μm)**		**Spectralis Foveal Thickness (μm)**	
**Patient #**	**Gender**	**Race**	**Age**	**OD**	**OS**	**OD**	**OS**
**1**	Female	Black	55	297 ± 0.6	286 ± 1.5	293 ± 1.0	279 ± 1.0
**2**	Female	White	28	250 ± 0.6	253 ± 1.2	255 ± 7.8	251 ± 0.6
**3**	Female	Black	49	273 ± 0.6	265 ± 1.0	275 ± 1.5	272 ± 0.6
**4**	Female	White	46	263 ± 0.6	262 ± 0.6	266 ± 2.1	268 ± 0.6
**5**	Male	White	57	287 ± 1.0	283 ± 1.2	287 ± 2.0	291 ± 3.5
**6**	Male	White	57	301 ± 1.0	308 ± 1.0	296 ± 2.1	305 ± 1.5
**7**	Male	White	36	313 ± 0.6	316 ± 0.6	309 ± 2.3	304 ± 4.6
**8**	Female	Asian	25	264 ± 1.0	262 ± 1.2	278 ± 5.5	271 ± 1.7
**9**	Male	White	83	312 ± 1.2	317 ± 0.6	317 ± 2.1	322 ± 1.7
**10**	Female	White	34	275 ± 0.0	269 ± 0.0	271 ± 0.0	264 ± 1.5
**Patients with Diseased Retinas**				**RAOCT Foveal Thickness (μm)**		**Spectralis Foveal Thickness (μm)**	
**11**	Female	White	91	284 ± 1.2	-	285 ± 3.5	-
**12**	Female	White	49	293 ± 1.5	295 ± 2.1	286 ± 1.2	291 ± 3.5
**13**	Female	White	60	300 ± 1.5	-	302 ± 4.6	-
**14**	Female	White	81	260 ± 2.1	228 ± 4.6	265 ± 3.8	228 ± 8.2
**15**	Female	Black	82	222 ± 1.2	-	224 ± 4.0	-
**16**	Male	White	69	-	304 ± 1.2	-	322 ± 9.3
**17**	Male	White	66	-	421 ± 1.7	-	430 ± 90.0
**18**	Female	White	81	572 ± 2.3	-	561 ± 17.2	-
**19**	Male	White	66	321 ± 0.6	280 ± 0.6	324 ± 17.2	276 ± 1.0
**20**	Male	White	76	-	286 ± 1.2	-	295 ± 3.5
**21**	Female	White	66	272 ± 0.6	283 ± 1.5	269 ± 5.1	285 ± 2.1
**22**	Female	Black	51	252 ± 1.2	249 ± 0.6	246 ± 2.1	246 ± 3.6
**23**	Female	Black	76	-	305 ± 1.5	-	326 ± 19.6
**24**	Male	White	71	474 ± 8.9	276 ± 2.0	454 ± 14.7	277 ± 5.6
**25**	Female	White	43	274 ± 4.4	361 ± 1.2	272 ± 2.1	373 ± 28.7

## Data Availability

The main data supporting the results in this work are available within the paper. The raw data acquired during the study are available from the corresponding author on reasonable request, subject to approval from the Duke University Medical Center Institutional Review Board.
